# Supplementary material for: Patterns of Intron Gain and Loss in Fungi
Source: PLoS Biol. 2004 Nov 30;2(12):e422. doi: 10.1371/journal.pbio.0020422 (PMC532390; doi:10.1371/journal.pbio.0020422)
Supplement: Table S1 — Also available at http://genes.mit.edu/NielsenEtAl/. (4.3 MB ZIP). [file pbio.0020422.st001.zip › NielsenEtAl/html/778.html]

AN5973.1.NCU07280.1.MG06599.1.FG05845.1


```
 CLUSTAL W (1.82) Multiple Sequence Alignments - Introns Inserted


Sequence 1: NCU07280.1	688 aa
Sequence 2: FG05845.1	630 aa
Sequence 3: MG06599.1	631 aa
Sequence 4: AN5973.1	640 aa
Alignment Length: 700 aa
Number Identitical Residues: 398 aa
Alignment Score (without introns) 18045


MG06599.1 	-----------------------------------MSWKLTKK1L--------~-KETHL
NCU07280.1	MAPTWTAAARTPARQANTPPTPARHLGILSILPSSPQWHLAVR~LPLCVEVPS0LKETHL
FG05845.1 	-----------------------------------MSWKLTKK1L--------~-KETHL
AN5973.1  	-----------------------------------MSWKLTKK1L--------~-KETHL
          	                                    .*:*: : *          *****

MG06599.1 	TTTLSNTFSRSPSTSTITDK--DDKSGDSGISGAATPTNE----~-------------SN
NCU07280.1	GPLSS-PFSRSPSTSTITDK--EDK-SQAGSSGAATPSNENTIA1ASEALTQAPIVKPPK
FG05845.1 	GQSLS-PFSRSPSTSTITDK--EKE-AQA--SGAVTPTTESAIA1ASEAMTQSPVVKPPK
AN5973.1  	APLTQ-TFTRSSSTSTIKGDSGEEAQAPAQTPTISSSASSNGIA1ASEALVSPP-VAPVK
          	    . .*:**.*****...:.:. .. :  .   :.:...  : ::.:  ...   . :

MG06599.1 	VG1ILVVTIHEGQGFSLPEQHRAAFGMGNQHSSSVSTGNGFGVAGSVRPGSSQKSPGVFN
NCU07280.1	PG~ILVVTLHEGSGFSLPEQYRNVFSS-NHGGNSMSTGSALNVAGSIRTNNSSRTANFLS
FG05845.1 	PG~ILVVTLHEGQGFSLPEQHRSAFAS-SHQG-SMSSGSAI--SGSVRPGSSQRTAGFSN
AN5973.1  	PG~ILIVTLHEGRSFALPQHYQQVFSSHIQNGYSMRP-----SSSSSHSTHSQSSSFVPS
          	 * **:**:*** .*:**:::: .*.   : . *: .      :.* :.  *. :. . .

MG06599.1 	G--RPQTSGGGFGTLPTNHGRFSGKYMPYALIDFDKVQVFVNSVEGSPDNPLWAGSNTQY
NCU07280.1	GSSRPQS-SGGFGAIPTNHGRISTKYMPYALIDFDKVQVFVNSVEGNPENPLWAGSNTQY
FG05845.1 	G--RPQSSAGGFNGIPTNHGRISGKYMPYALLDFDKVQVFVNSVDGNPENPLWAGGNTQY
AN5973.1  	N--RPQSTSSGINAAPTIHGRYSTKYLPYALLDFDKNQVFVDAVSGTPENPLWAGDNTAF
          	.  ***::..*:.  ** *** * **:****:**** ****::*.*.*:******.** :

MG06599.1 	KFDVSRVTELTIHLYIRNPNAPPGSGRSQDIFLGVCRIQPRFEER-QPFVEDPKMSKKDK
NCU07280.1	KFDVSRVTELAVHLYIRNPNAAPGSGRSQDIFLGVVRINPRFEEK-QQFVDDPKASKKDR
FG05845.1 	KFDVSRVTELVIHLYMRNPTAPPGSGRSQDIFLGVVRINPRFEER-QQYVEDPKASKKDR
AN5973.1  	KFDVSRKTDLNVQLYLRNPAARPGAGRNDDIFLGAVKVHPRFEEEIQPYIDDPKLSKKDN
          	****** *:* ::**:*** * **:**.:*****. :::*****. * :::*** ****.

MG06599.1 	EKAQAEWAKRERALGFSGVEWADVQYGTGRLKIGVEFVANRTGKLKLDEDFELLKVVGKG
NCU07280.1	EKAAADFANKERALGHSGVEWVDVQYGTGKLKIGVEYVENRAGKLKI-EDFELLKVVGKG
FG05845.1 	EKAAAEFAAR---HGHQGAEWVDVQYGTGKIKVGVDYVETRAGKLKI-EDFELLKLVGKG
AN5973.1  	QKAAAAHAEQERALGQVGAEWHDLQFGSGSLKISVSFVESKHRSLKL-EDFDLLKVVGKG
          	:** *  * :. : *  *.** *:*:*:* :*:.*.:* .:  .**: ***:***:****

MG06599.1 	SFGKVMQVR~KKDTNRIYALKTIRKAHIISRSEVTHTLAERSVLAQINNPFIVPLKFAFQ
NCU07280.1	SFGKVMQVR~KKDTNRIYALKTIRKAHIISRSEVAHTLAERSVLAQINNPFIVPLKFTFQ
FG05845.1 	SFGKVMQVR~KKDTNRIYALKTIRKAHIISRSEVAHTLAERSVLAQINNPFIVPLKFSFQ
AN5973.1  	SFGKVMQVM2KKDTGRIYALKTIRKAHIISRSEVTHTLAERSVLAQINNPFIVPLKFSFQ
          	********  ****.*******************:**********************:**

MG06599.1 	SPEKLYFVLAFVNGGELFHHLQKEQRFDVNRSRFYTAELICALECLHGFNVVYRDLKPEN
NCU07280.1	SPEKLYFVLAFVNGGELFHHLQKEQRFDVNRSRFYTAELLCALECLHGFNVIYRDLKPEN
FG05845.1 	SPEKLYFVLAFVNGGELFHHLQKEHRFDVNRSRFYTAELLCALECLHGFSVIYRDLKPEN
AN5973.1  	SPEKLYFVLAFVNGGELFHHLQREQRFDVNRARFYTAELLCALECLHGFKVIYRDLKPEN
          	**********************:*:******:*******:*********.*:********

MG06599.1 	ILLDYQGHIALCDFGLCKLDMKDEDHTNT~FCGTPEYLAPELLEGKGYDKAVDWWTLGVL
NCU07280.1	ILLDYQGHIALCDFGLCKLDMKDEDRTNT~FCGTPEYLAPELLMGNGYNKTVDWWTLGVL
FG05845.1 	ILLDYQGHIALCDFGLCKLDMKDEDRTNT~FCGTPEYLAPELLMGQGYNKTVDWWTLGVL
AN5973.1  	ILLDYTGHIALCDFGLCKLDMKDEDRTNT1FCGTPEYLAPELLLGQGYTKTVDWWTLGVL
          	***** *******************:*** ************* *:** *:*********

MG06599.1 	LYEMLTGLPPFYDENTNEMYKKILYQPLDFPGPEVVPPAAQDLLRKLLDRDPKTRLGSPE
NCU07280.1	LYEMLTGLPPFYDENTNEMYRKILSEPLHFPGPEVVPPAAKDLLTKLLNRDPQQRLG---
FG05845.1 	LYEMLTGLPPFYDENTNEMYRKILSEPLHFS--DVVPPAAKDLLTKLLNRNPEERLG---
AN5973.1  	LYEMLTGLPPFYDENTNDMYRKILQEPLTFPSSDIVPPAARDLLTRLLDRDPNRRLG---
          	*****************:**:*** :** *...::*****:*** :**:*:*: ***   

MG06599.1 	RGRGAAEIKSHPFFHAIDWRKLLQRKYEPAFKPSV0ANALDTTNFDPEFTSEAPQDSYVE
NCU07280.1	-ANGAAEIKAHPFFHAIDWRKLLQRKYEPAFKPNV0VDALDTANFDPEFTSELPQDSYVE
FG05845.1 	-ANGSAEIKAHPFFHAIDWRKLLQRKYEPTFKPSV0ADALDTTNFDPEFTSEAPQDSFVD
AN5973.1  	-ANGAAEIKSHHFFANIDWRKLLQRKYEPSFRPNV0ADARDTKNFDAEFTSEAPKDSYVD
          	 ..*:****:* **  *************:*:*.* .:* ** ***.***** *:**:*:

MG06599.1 	GPVLSQTMQDQFQGFSYNRPIAGLDDAGGSVKDPSFVGSLQGGR---
NCU07280.1	GPILSETMQNQFTGFSYNRPIAGLGDAGGSVKDPSFASSLQDNSSRR
FG05845.1 	GPMLSQTMQNQFQGFSYNRPIAGLGDAGGSVKDPSFVGSLTDNR---
AN5973.1  	GPMLSSTQQQQFEGWSYNRPVAGLGDAGGSVKDPSFASIPEDSRF--
          	**:**.* *:** *:*****:***.***********..   ..
```
